# Supplementary material for: Modeling glioblastoma heterogeneity as a dynamic network of cell states
Source: Mol Syst Biol. 2021 Sep 16;17(9):e10105. doi: 10.15252/msb.202010105 (PMC8444284; doi:10.15252/msb.202010105)
Supplement: Supplementary file 6 — Source Data for Figure 5 [file MSB-17-e10105-s004.zip › Figure5A_sourcedata/GSEA_3071/hallmarks_stateIII.GseaPreranked.1623742540963/HALLMARK_IL2_STAT5_SIGNALING.html]

Details for gene set HALLMARK\_IL2\_STAT5\_SIGNALING[GSEA]

|  || Dataset | state33071 |
| Phenotype | NoPhenotypeAvailable |
| Upregulated in class | na\_neg |
| GeneSet | HALLMARK\_IL2\_STAT5\_SIGNALING |
| Enrichment Score (ES) | -0.37913376 |
| Normalized Enrichment Score (NES) | -2.1147883 |
| Nominal p-value | 0.0 |
| FDR q-value | 0.0034852354 |
| FWER p-Value | 0.028 |
Table: GSEA Results Summary

  

Fig 1: Enrichment plot: HALLMARK\_IL2\_STAT5\_SIGNALING      
 Profile of the Running ES Score & Positions of GeneSet Members on the Rank Ordered List

  

| PROBE | GENE SYMBOL | GENE\_TITLE | RANK IN GENE LIST | RANK METRIC SCORE | RUNNING ES | CORE ENRICHMENT || 1 | IKZF2 |  |  | 430 | -0.250 | -0.1019 | No |
| 2 | HK2 |  |  | 432 | -0.250 | -0.0929 | No |
| 3 | HIPK2 |  |  | 546 | -0.257 | -0.1125 | No |
| 4 | PHLDA1 |  |  | 589 | -0.259 | -0.1138 | No |
| 5 | MYC |  |  | 599 | -0.260 | -0.1064 | No |
| 6 | PRKCH |  |  | 603 | -0.260 | -0.0976 | No |
| 7 | CCNE1 |  |  | 677 | -0.263 | -0.1067 | No |
| 8 | MAFF |  |  | 735 | -0.266 | -0.1115 | No |
| 9 | PLAGL1 |  |  | 783 | -0.268 | -0.1137 | No |
| 10 | RHOB |  |  | 865 | -0.272 | -0.1245 | No |
| 11 | GALM |  |  | 1150 | -0.287 | -0.1873 | No |
| 12 | TTC39B |  |  | 1193 | -0.290 | -0.1874 | No |
| 13 | TIAM1 |  |  | 1276 | -0.294 | -0.1976 | No |
| 14 | PRNP |  |  | 1309 | -0.295 | -0.1949 | No |
| 15 | IGF2R |  |  | 1432 | -0.302 | -0.2153 | No |
| 16 | WLS |  |  | 1517 | -0.307 | -0.2256 | No |
| 17 | KLF6 |  |  | 1596 | -0.312 | -0.2342 | No |
| 18 | CD83 |  |  | 1729 | -0.321 | -0.2564 | No |
| 19 | NT5E |  |  | 1769 | -0.323 | -0.2545 | No |
| 20 | BMPR2 |  |  | 1779 | -0.323 | -0.2448 | No |
| 21 | P2RX4 |  |  | 1861 | -0.328 | -0.2535 | No |
| 22 | NFKBIZ |  |  | 1903 | -0.330 | -0.2519 | No |
| 23 | SMPDL3A |  |  | 1917 | -0.330 | -0.2430 | No |
| 24 | SPRY4 |  |  | 1970 | -0.334 | -0.2440 | No |
| 25 | FAH |  |  | 2007 | -0.335 | -0.2409 | No |
| 26 | MYO1E |  |  | 2017 | -0.337 | -0.2307 | No |
| 27 | MXD1 |  |  | 2053 | -0.338 | -0.2272 | No |
| 28 | XBP1 |  |  | 2097 | -0.341 | -0.2256 | No |
| 29 | SLC2A3 |  |  | 2117 | -0.343 | -0.2178 | No |
| 30 | CD44 |  |  | 2471 | -0.371 | -0.2953 | No |
| 31 | CKAP4 |  |  | 2664 | -0.387 | -0.3305 | No |
| 32 | ALCAM |  |  | 2853 | -0.403 | -0.3641 | Yes |
| 33 | ITGA6 |  |  | 2899 | -0.408 | -0.3606 | Yes |
| 34 | LIF |  |  | 2908 | -0.409 | -0.3475 | Yes |
| 35 | FURIN |  |  | 2932 | -0.412 | -0.3381 | Yes |
| 36 | TGM2 |  |  | 2933 | -0.413 | -0.3228 | Yes |
| 37 | ITGAV |  |  | 2942 | -0.414 | -0.3094 | Yes |
| 38 | CD81 |  |  | 2945 | -0.414 | -0.2946 | Yes |
| 39 | BHLHE40 |  |  | 2981 | -0.417 | -0.2881 | Yes |
| 40 | SCN9A |  |  | 2996 | -0.420 | -0.2761 | Yes |
| 41 | EMP1 |  |  | 2998 | -0.420 | -0.2608 | Yes |
| 42 | IKZF4 |  |  | 3023 | -0.423 | -0.2513 | Yes |
| 43 | CCND2 |  |  | 3037 | -0.425 | -0.2389 | Yes |
| 44 | BCL2 |  |  | 3081 | -0.429 | -0.2340 | Yes |
| 45 | ADAM19 |  |  | 3132 | -0.435 | -0.2308 | Yes |
| 46 | APLP1 |  |  | 3156 | -0.439 | -0.2204 | Yes |
| 47 | BMP2 |  |  | 3307 | -0.462 | -0.2421 | Yes |
| 48 | PLEC |  |  | 3324 | -0.466 | -0.2289 | Yes |
| 49 | SYNGR2 |  |  | 3356 | -0.472 | -0.2194 | Yes |
| 50 | TNFSF10 |  |  | 3485 | -0.502 | -0.2338 | Yes |
| 51 | LRRC8C |  |  | 3500 | -0.507 | -0.2186 | Yes |
| 52 | NRP1 |  |  | 3562 | -0.532 | -0.2146 | Yes |
| 53 | RGS16 |  |  | 3616 | -0.555 | -0.2076 | Yes |
| 54 | ITIH5 |  |  | 3619 | -0.560 | -0.1873 | Yes |
| 55 | F2RL2 |  |  | 3645 | -0.574 | -0.1725 | Yes |
| 56 | AHNAK |  |  | 3660 | -0.584 | -0.1544 | Yes |
| 57 | CSF1 |  |  | 3669 | -0.590 | -0.1345 | Yes |
| 58 | RORA |  |  | 3716 | -0.630 | -0.1230 | Yes |
| 59 | SYT11 |  |  | 3748 | -0.669 | -0.1061 | Yes |
| 60 | TNFRSF21 |  |  | 3763 | -0.691 | -0.0841 | Yes |
| 61 | COL6A1 |  |  | 3863 | -0.872 | -0.0773 | Yes |
| 62 | PTCH1 |  |  | 3883 | -1.016 | -0.0444 | Yes |
| 63 | SPP1 |  |  | 3920 | -1.508 | 0.0023 | Yes |
Table: GSEA details [plain text format]

  

Fig 2: HALLMARK\_IL2\_STAT5\_SIGNALING: Random ES distribution      
 Gene set null distribution of ES for **HALLMARK\_IL2\_STAT5\_SIGNALING**
